# AFM Imaging Reveals Topographic Diversity of Wild Type and Z Variant Polymers of Human α_1_-Proteinase Inhibitor

**DOI:** 10.1371/journal.pone.0151902

**Published:** 2016-03-23

**Authors:** Maria Gaczynska, Przemyslaw Karpowicz, Christine E. Stuart, Malgorzata G. Norton, Jeffrey H. Teckman, Ewa Marszal, Pawel A. Osmulski

**Affiliations:** 1 Department of Molecular Medicine, University of Texas Health Science Center at San Antonio, San Antonio, Texas, United States of America; 2 Center for Biologics Evaluation and Research, U.S. Food and Drug Administration, Silver Spring, Maryland, United States of America; 3 Department of Pediatrics and Biochemistry, Saint Louis University School of Medicine, Cardinal Glennon Children’s Medical Center, St. Louis, Missouri, United States of America; University of Pittsburgh School of Medicine, UNITED STATES

## Abstract

α_1_-Proteinase inhibitor (antitrypsin) is a canonical example of the serpin family member that binds and inhibits serine proteases. The natural metastability of serpins is crucial to carry out structural rearrangements necessary for biological activity. However, the enhanced metastability of the mutant Z variant of antitrypsin, in addition to folding defect, may substantially contribute to its polymerization, a process leading to incurable serpinopathy. The metastability also impedes structural studies on the polymers. There are no crystal structures of Z monomer or any kind of polymers larger than engineered wild type (WT) trimer. Our understanding of polymerization mechanisms is based on biochemical data using in vitro generated WT oligomers and molecular simulations. Here we applied atomic force microscopy (AFM) to compare topography of monomers, in vitro formed WT oligomers, and Z type polymers isolated from transgenic mouse liver. We found the AFM images of monomers closely resembled an antitrypsin outer shell modeled after the crystal structure. We confirmed that the Z variant demonstrated higher spontaneous propensity to dimerize than WT monomers. We also detected an unexpectedly broad range of different types of polymers with periodicity and topography depending on the applied method of polymerization. Short linear oligomers of unit arrangement similar to the Z polymers were especially abundant in heat-treated WT preparations. Long linear polymers were a prominent and unique component of liver extracts. However, the liver preparations contained also multiple types of oligomers of topographies undistinguishable from those found in WT samples polymerized with heat, low pH or guanidine hydrochloride treatments. In conclusion, we established that AFM is an excellent technique to assess morphological diversity of antitrypsin polymers, which is important for etiology of serpinopathies. These data also support previous, but controversial models of in vivo polymerization showing a surprising diversity of polymer topography.

## Introduction

Serpins belong to a superfamily of several hundred highly conserved, structurally homologous proteins with a metastable native structure [1–3]. The intrinsic metastability of serpins constitutes a basis of their major and best-known biological function as regulators of serine and cysteine protease cascades [4,5]. Unfortunately, the metastability makes serpins structurally prone to mutation-induced polymerization that leads to their inactivation [3]. The polymerization prone protease inhibitors from the serpin superfamily are often referred to as “canonical” serpins. Besides causing serpin deficiency, intracellular accumulation of these misfolded proteins and their polymers is toxic. The diseases with serpin-related etiology are collectively known as serpinopathies [6]. The most common serpinopathy is related to the canonical and most abundant serpin, α_1_-proteinase inhibitor (α_1_-PI) also known as alpha-1-antitrypsin (α_1_-AT), and affects patients’ lungs or liver in “loss of function”or “toxic gain of function” fashions, respectively [7,8]. α_1_-PI is produced abundantly in a liver but also in lungs and gut epithelial cells. It is then secreted into the bloodstream, and distributed throughout the body. Its primary target is neutrophil proteases, which non-specifically leak from granulocytes during phagocytosis. The key role of secreted α_1_-PI is to protect tissues, lungs in particular, from damage during inflammation episodes [9]. Individuals with greatly reduced serum levels of α_1_-PI suffer from progressive emphysema [10]. This deficiency most often results from the Glu-342-Lys mutation (the so called Z variant) and when present in the homozygous ZZ state, it accounts for the majority of disease cases in humans. The substitution triggers a strong propensity of mutant α_1_-PI to polymerize [3,11]. In turn, the Z variant polymers are poorly secreted from hepatocytes, where about 85% of α_1_-PI is retained in endoplasmic reticulum and overloads protein quality control machinery [8]. The accumulation of polymerized Z variant in the hepatocytes is associated with an increased risk of chronic liver disease, cirrhosis and liver cancer [3,12].

Despite extensive efforts, the molecular mechanism underlying serpin polymerization is far from complete understanding. The crystal structure of wild type (WT) α_1_-PI monomer has been solved (1qlp) [13]. The critical part of the conserved structure of serpins consists of the mobile and exposed reactive center loop (RCL), and the set of three β sheets (A, B, C) and nine α helices. The target protease cleaves the loop prompting the N-terminal part of the loop to insert into A β sheet of the same molecule, which stabilizes the structure. It has been postulated that positioning of the Z mutation at the base of RCL promotes structural metastability and intermolecular loop-sheet insertion; however the structural details of the process are a matter of debate [3,14,15]. Not only the actual structure of native serpin polymers remains unknown, but also the monomer of disease-relevant Z variant has escaped crystallization efforts. Several proposed models of polymerization have been based on molecular modeling and crystal structure analysis of dimers or trimers formed *in vitro* from proteolytically cleaved or mutated and exposed to elevated temperatures serpin molecules [16,17]. Similarly, biochemical characterization of oligomers was limited to particles formed *in vitro* from the monomers partially denatured by elevated temperature, low pH or guanidine hydrochloride (GuHCl) [18]. The oligomers and large *in vivo* formed polymers of the Z variant were imaged by electron microscopy, however no further analysis of the images of relaxed, uniform “beads on the string” particles was performed [15,19]. Nevertheless, a small molecule mimicking the RCL and thus blocking the loop-sheet insertion and preventing polymerization *in vitro* have been recently developed [20]. In another important attempt to block polymerization, a monoclonal antibody fragment was found to stabilize the Z variant monomer while preserving its antiproteinase activity *in vitro* and in cell culture [21].

Here we propose a novel approach to structural characterization of α_1_-PI using atomic force microscopy (AFM). To the best of our knowledge, no canonical serpin molecules have been imaged by AFM before. We present images of purified human antitrypsin monomers, *in vitro* formed WT oligomers and *in vivo* formed Z mutant polymers. The initial analysis of images allowed us to acknowledge differences between WT and Z variant particles and to directly assess structural diversity of oligomers and polymers.

## Material and Methods

### Antitrypsin monomers and polymers

The human wild type (WT) α_1_-PI monomer, prepared from pooled plasma, was a gift from CSL Behring. The preparation was subjected to chromatography as previously described [13]. The 500μM sample was diluted in 20 mM sodium phosphate buffer pH 7.4 with 130 mM NaCl. Based on HPLC fractionation, the preparation was stable to the storage conditions (-80°C), and contained minute amount of dimers. The preparation of human Z variant monomer was graciously provided by Dr. Mark Brantly from the University of Florida, Gainesville, FL. The content of monomers and larger species in 2.7 μM preparations was verified by native PAGE (7.5% acrylamide; standard Laemmli system) and the partition of forms was approximated from Coomassie or silver stained gels with Image J. The PAGE verification was performed before each use of the preparations stored at -20°C or -80°C, however no significant changes in partition of forms was noted upon months of storage. The wild type monomers were *in vitro* polymerized with elevated temperature (55°C, 4 hrs of incubation at pH = 7.4), low pH (pH = 4.1, 2 hrs of incubation at 25°C) or 1.4M GuHCl (37.5 hrs, 25°C, pH = 7.4). Human Z mutant *in vivo* formed polymers were isolated from the liver of a transgenic mouse expressing human Z variant α_1_-PI (PiZ mouse) maintained on a C57BL/6 background, the mouse strain commonly used as a model for serpinopathy studies [22–25]. The PiZ mouse has been comprehensively characterized and shown that it closely recapitulates disease manifestation in humans including Z variant expression and accumulation in liver, evidence of numerous histopathological globular inclusions, increased hepatocellular proliferation and development of hepatocellular carcinoma [23,26–28]. The human Z variant polymers formed in transgenic mouse liver were isolated using the procedure developed by An et al. [22], slightly modified. In short, the tissue added to the extraction buffer (50 mM Tris HCl pH 8, 150 mM NaCl, 5 mM KCl, 5 mM MgCl_2_, 0.5% Triton X-100, and protease inhibitor cocktail) was disintegrated in a prechilled Dounce homogenizer and by passing through a 28-gouge needle. The homogenate was centrifuged at 10,000xg for 30 min at 4°C. The supernate (soluble fraction) was removed, the pellet was washed with reconstitution buffer (20 mM sodium phosphate pH 7, 130 mM NaCl, 10 mM EDTA) sonicated on ice using a Branson Sonifier 450, and used for AFM imaging. The insoluble fraction contained nearly exclusively Z variant α_1_-PI, as assessed by SDS-PAGE and Western blotting (S1 Fig).

### Atomic Force Microscopy

AFM imaging was performed in tapping (oscillation) mode in air, with a Nanoscope IIIa microscope (Bruker Corp.). The antitrypsin preparations were diluted in buffer (2 mM Na phosphate, 13 mM NaCl, pH 7.4) to the concentration of 20–100 nM. A 3μl drop of the solutions was deposited on a freshly cleaved muscovite mica (Ted Pella, Inc.) surface. The samples were immediately briefly spun on a turntable to prevent clumping or collapsing of polymers. After 3 min of incubation at room temperature to allow for electrostatic attachment of most of the particles to mica surface, the mica was washed with double distilled water and briefly dried under a stream of ultra pure nitrogen [29]. Imaging was carried out with TESP probes (Bruker Corp.), with resonant frequency tuned to 280–320 kHz, the amplitude of 30–100 mV, the setpoint between 1.4 and 1.8 V, and scan rate of 2.5–3.0 Hz. The relatively high setpoint assured gentle, low-force imaging. Wild type and mutant preparations in most cases were scanned with the same probes, back–to—back. The radii of curvature of the tips were approximated to measure about 7 nm based on imaging of colloidal gold standard (Ted Pella, Inc.). Images of areas ranging from 500 x 500 nm to 2 x 2 μm were collected in a height mode with digital resolution of 512 x 512 pixels. For each experiment at least ten separate fields with particles were imaged, in trace and retrace directions, and selected fields were repeatedly scanned at least three times to test stability of particle attachment to mica and reproducibility of imaging. The images were subjected to a standard flattening (order 1) and plane-fitting, supplemented by occasional removal of scan lines-streaks in the Nanoscope software (v. 5.12; Bruker Corp.). No other processing was applied to the raw images subjected to particle analysis. For display, the brightness and contrast was adjusted with the Nanoscope software.

### Morphometric and statistical analysis

The grain analysis function in the SPIP software (v. 5.1, Image Metrology, Denmark) was used to count the particles in monomer preparations, to collect their metrics, and to approximate the lengths (“fiber length” parameter) of polymer particles [29]. The grains were detected automatically with a preset condition to exclude very large amorphous objects. Such objects are routinely present in images of biomolecules and constitute less than 5% of total number of particles. Majority of images used for calculations were scanned with a pixel size of about 1.5 nm (750 nm x 750 nm fields). The topmost parts of particle images, to the depths of up to 1 nm, where tip broadening is likely minimal, were analyzed. Such approach prevented automatic classification of nearby single molecules as one large particle due to the tip broadening phenomenon prominent in lower regions of the height mode image. The full height of the particles was approximated by section analysis (Nanoscope software) of the edges of occasionally found closely packed patches of wild type molecules [30]. Tips of uniform radii were selected for analysis, which was performed on raw parameters. Descriptive statistics was carried out with OriginPro 8.5 (OriginLab Corp., Mass., USA) and Excel of MS Office 2003. The data distribution and frequency was tested with OriginPro 8.5. The approximation of length of units in linear polymers was carried out with the section analysis of the Nanoscope software. The distances between lowest points of the wave-shaped lateral sections through randomly chosen straight fragments of the polymer fibrils were computed and presented as the approximate unit length.

Volume of a WT serpin monomer based on its crystal structure (pdb: 1qlp) was calculated with VADAR v. 1.8 (Volume Area Dihedral Angle Reporter, http://vadar.wishartlab.com; University of Alberta, Edmonton, Canada) using the Standard Voronoi procedure where the Shrake method was applied to calculate Van der Waals radii and to assess the solvent accessible surface area. Additionally, the volume was calculated with 3V (Voss Volume Voxelator: http://3vee.molmovdb.org) volume assessor by rolling a virtual probe over the 1qlp protein surface [31,32]. The probe radius of 1.4, 3 and 10 Å was applied to obtain the solvent-excluded volume and protein shell volume with or without cavities. Simulated AFM topography of the 1qlp protein was generated with the AFMSim module of Microscope Simulator v. 1.3.1 (Center for Computer Integrated Systems for Microscopy and Manipulation at the University of North Carolina Chapel Hill, National Institute of Biomedical Imaging and Bioengineering Resource). The AFM surface model was used to calculate the serpin volume with an Image Statistics function of the Simulator. The calculated volume included an additional component resulting from the AFM tip geometry resulting in a systematic volume enlargement.

## Results

### AFM renders an accurate topography of the α_1_-PI monomers

**Fig 1:**
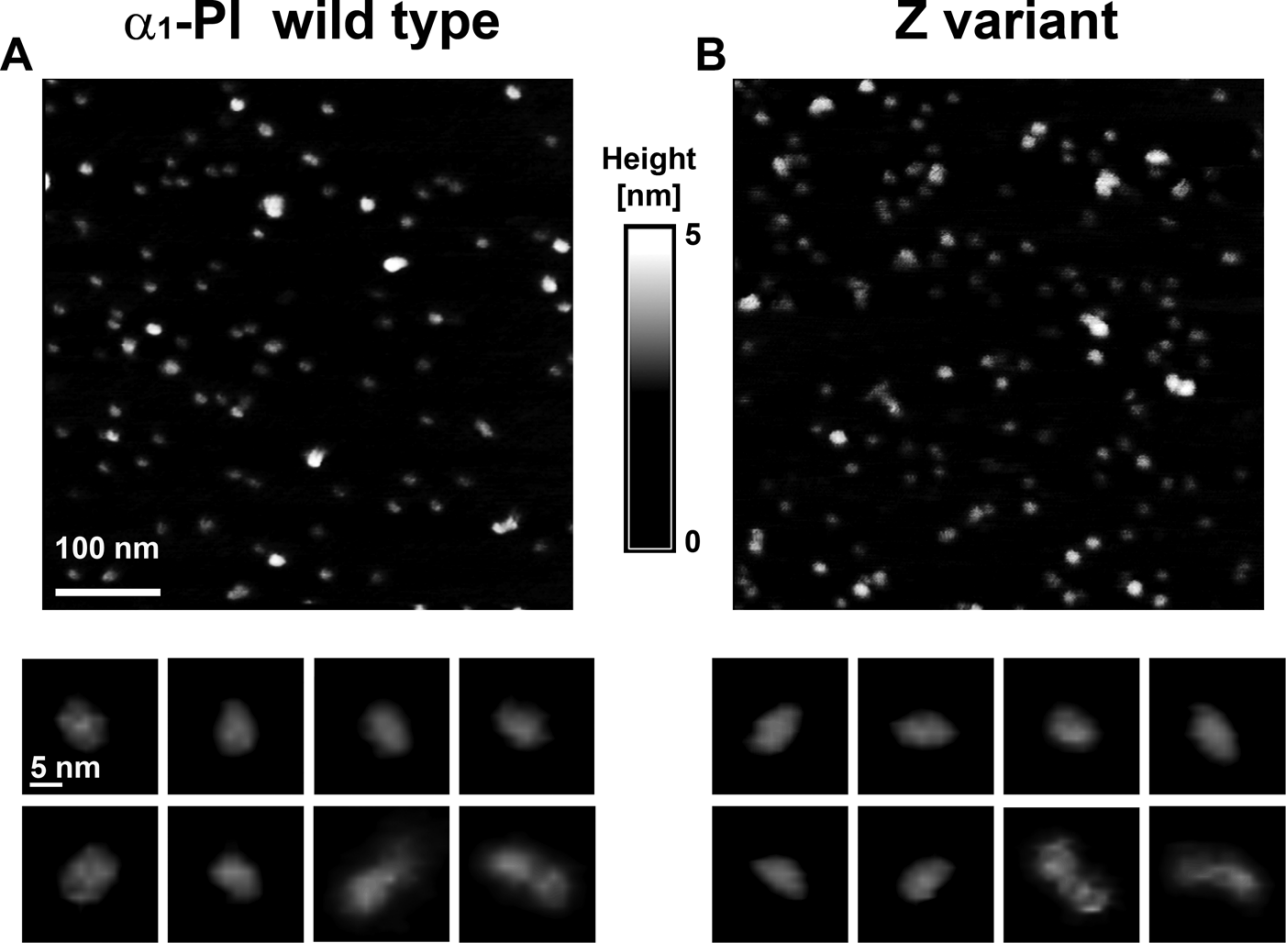
AFM height images show topographic diversity of human wild type and Z variant α_1_-PI. Images of wild type α_1_-PI (antitrypsin) (A) and Z variant α_1_-PI (B) monomer preparations. The shades-of-grey height scale presented here has been consistently used in Figs 2A, 4 and 5. Each top image shows an AFM field of particles, whereas arrays of zoomed-in images of typical α_1_-PI molecules are presented at the bottom. The height images were collected with the tapping (oscillating) mode in air. Majority of the α_1_-PI molecules were oblong showing the dimensions consistent with the crystal structure models of WT monomers [15]. However, a minor but significant fraction was clearly built from two monomeric units, as can be seen in the last two zoomed-in panels in A and B galleries.

We established conditions for reproducible AFM imaging of unfixed antitrypsin molecules. Fig 1 shows representative fields of WT (A) and Z mutant (B) particles randomly distributed on mica. Under the conditions used for most of the imaging, there were 100–200 particles detected per square micrometer. This relatively low object density was selected to minimize the possibility of crowding-induced, non-specific interactions between molecules.

The location and appearance of particles did not change during repeated scanning of the same fields, and did not significantly differ in trace and retrace images, assuring that the attachment of particles to mica was stable to the scanning conditions. To evaluate temporal stability of the α_1_-PI monomers, we compared a distribution of particle volumes measured with the SPIP software (see below). Identical histograms of volume frequency were obtained with the same batches of monomers within a span of several months. There were no additional long, strand-shaped particles, or other types of polymers detected even after a prolonged storage of the samples indicating that spontaneous oligomerization was insignificant in the stored monomer preparations.

A majority of imaged particles were fairly uniform. They were visualized mostly as oblong objects (Fig 1, gallery of zoomed-in portraits of the particles). A qualitative comparison did not reveal major topographical differences between WT and Z monomers (compare Fig 1 bottom A and B). In addition to the prevalent oval molecules, occasional larger particles of diverse shapes were observed. Most of them were clearly built from two units, strongly suggesting that they depict dimers (Fig 1, last two panels in zoom-ins galleries in A and B). From among about two thousands of visually inspected objects, several cases of up to five-unit oligomeric particles were noted as well, however these were the largest presumed oligomers detected.

**Fig 2:**
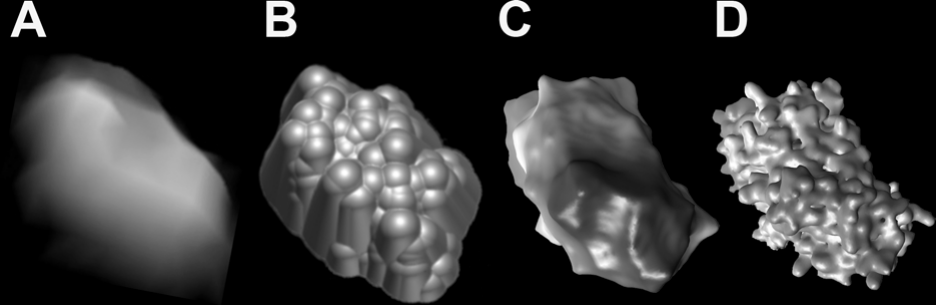
Comparison of α_1_-PI topography generated with AFM, the AFM simulator and the crystal structure model. The AFM generated topography of the wild type α_1_-PI is in a good agreement with the shape and dimensions of particles obtained with the AFM simulator and calculated from a model of the protein surface occluded by a water shell. In contrast, the waterless Van der Waals protein surface model was too rich in structural details to be efficiently compared with AFM topographs. The models were based on the crystal structure of α_1_-PI WT monomer (PDB ID: 1qlp [31]). The length of a space-filled crystal structure model of the kidney-shaped antitrypsin molecule was 7–8 nm and its width up to 4–5 nm. (A) a tilted AFM image (side plot) of a typical WT monomer particle; (B) a surface model generated with the Microscope Simulator using the cone-sphere tip model of a radius 7 Å and a cone angle 25°; (C) a Van der Waals protein surface model generated with the 3V program using a probe of 10 Å radius that adds the water shell; (D) a protein surface model obtained as in (C) by applying a probe radius of 0 Å (“waterless”). Models presented in B-D are structurally aligned.

To get a better understanding of expected morphology of WT serpin monomers we compared topography of antitrypsin imaged by AFM to molecular surface models generated from its crystal structure (Protein Data Bank ID: 1qlp) using different simulation methods. To perform the AFM simulation we selected a single orientation of the monomer, expected to lie flat on the mica surface and to produce images of oblong objects. The results are presented in Fig 2. The topography of α_1_-PI detected with AFM (Fig 2A) resembled a surface created with the AFM simulator (Fig 2B). Despite matching the tip geometry used for the imaging and for the simulation, the AFM simulator produced a more detailed model than the probe could detect by scanning the unfixed protein. The phenomenon of blurring the images could be attributed to resolution-limiting thermal motions of the molecules. Also, we may expect that a water shell occluding the structural details is present on the surface of gently dried protein particles. Such water shell was not incorporated in the AFM simulation model in Fig 2B. In contrast, the 3D generated model (Fig 2C) included a 10Å thick water layer over the van der Waals surface of 1qlp and delivered a rounded particle with a shape strongly resembling the actual AFM images presented in Fig 1 zoom-ins and in Fig 2A. Finally, analysis of the shape of 3V created “waterless” van der Waals surface of 1qlp (Fig 2D) confirmed that the general topography of the antitrypsin molecule was accurately rendered by AFM with the expected loss of structural details.

To enable future studies on the multiplicity of monomers that form polymers and to compare metrics of the particles, we focused on the volume of antitrypsin. The volume of AFM detected molecules was determined with a grain analysis function of the SPIP software, whereas volume of the monomer models was calculated with the AFM simulator, VADAR software, and 3V software. As noted above, the water-occluded van der Waals surface model (Fig 2C) provided the best approximation of the shape of AFM images of antitrypsin monomers (molecule example: Fig 2A). The volume of the model illustrated in Fig 2C was 72 nm^3^ very similar to the approximated volume of the single particle presented in Fig 2A (75 nm^3^). The volume of a simulated AFM model was approximately 91 nm^3^ (Fig 2B), with the enlargement attributed to the applied cone-sphere probe shape leading to an exaggerated tip broadening effect. On the other hand, the van der Waals volume of the 1qlp based model (Fig 2D) was approximately 40.3 nm^3^ with the 3V (0.5A voxel) and 53.8 nm^3^ with the VADAR (total volume with packing) software. The discrepancy between the two values likely resulted from the different algorithms used for the calculations and the way volumes of cavities were estimated in both programs. Both values were significantly lower than the experimental volume. However, such discrepancy between the crystal structure-based and experimental volumes was anticipated since the former estimation did not incorporate water shell, dynamic blurring, or tip broadening effects.

**Fig 3:**
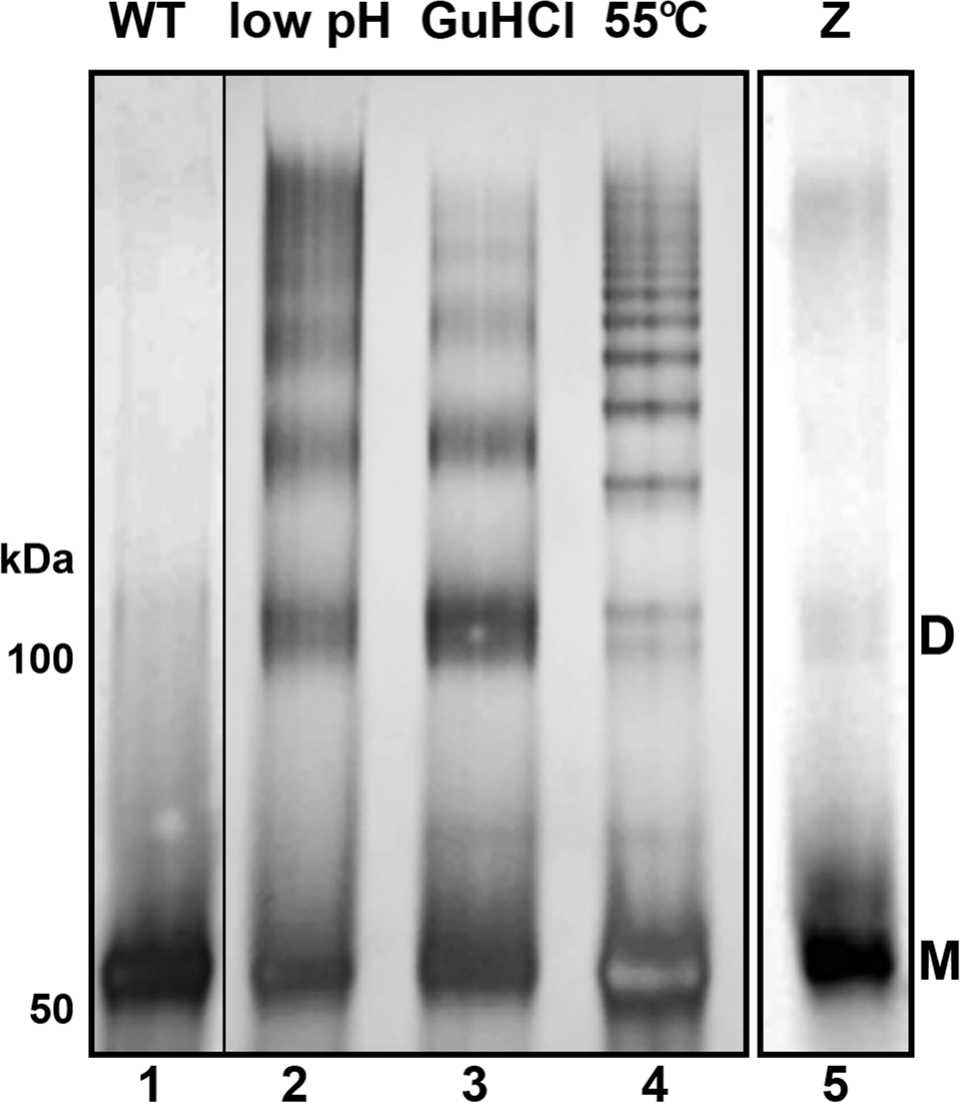
Native PAGE analysis of α_1_-PI monomers and *in vitro* formed polymers. Polymers were formed from purified WT plasma-derived α_1_-PI monomers (lane 1) by incubation at pH 4.1 (lane 2), incubation with 1.4M guanidinium chloride (lane 3) or incubation at 55°C (lane 4). Lane 5 presents purified human Z variant monomer. Quantification of the bands indicated that approximately 87% of the protein was migrating as a monomer in lane 1 (WT), markedly more than in lane 5 (69%; Z variant). M–monomer; D–dimer. Approximated molecular weights are indicated on the left. Lanes 2 to 4 were run concurrently on the same gel.

Figs 1 and 2A presented images of single antitrypsin molecules. Particles larger than typical monomers were detectable in both WT and Z variant preparations (Fig 1), however they were rather infrequent in the former. The observation was consistent with the data obtained with the non-denaturing PAGE (Fig 3). While about 70% of protein migrated as monomer in the Z variant preparation, the content of monomer was close to 90% in the WT preparation (Fig 3, lanes 1 and 5). Four distinct preparations of the WT α_1_-PI contained 91%±1% of monomers, as approximated from the electrophoregrams. Since we were interested in morphology of not only monomers but also spontaneously formed larger species, we attempted to enrich the WT preparation in oligomers for the purpose of AFM imaging. We noticed that washing of the WT particles deposited on the AFM substrate, routinely used before drying and- imaging, served the purpose well. We used mean volume of all particles imaged and evaluated by grain analysis in several fields as a rough indicator of relative content of larger particles. After a single wash the mean volume was 68 nm^3^ (n = 133 particles). After two washes, the mean volume increased to 86 nm^3^ (n = 46). After triple-wash, often used as a standard procedure, the mean volume was at 91 nm^3^ (n = 847), clearly indicating increased contribution of oligomeric particles. Interestingly, when Z variant preparations were submitted to the same procedure, the mean volumes remained relatively stable: with 96 nm^3^ (n = 157 particles) after a single wash, 108 nm^3^ (n = 130) after two washes and 94 nm^3^ (n = 639) after three washes. Therefore, we used triple-washed preparations in subsequent imaging.

**Fig 4:**
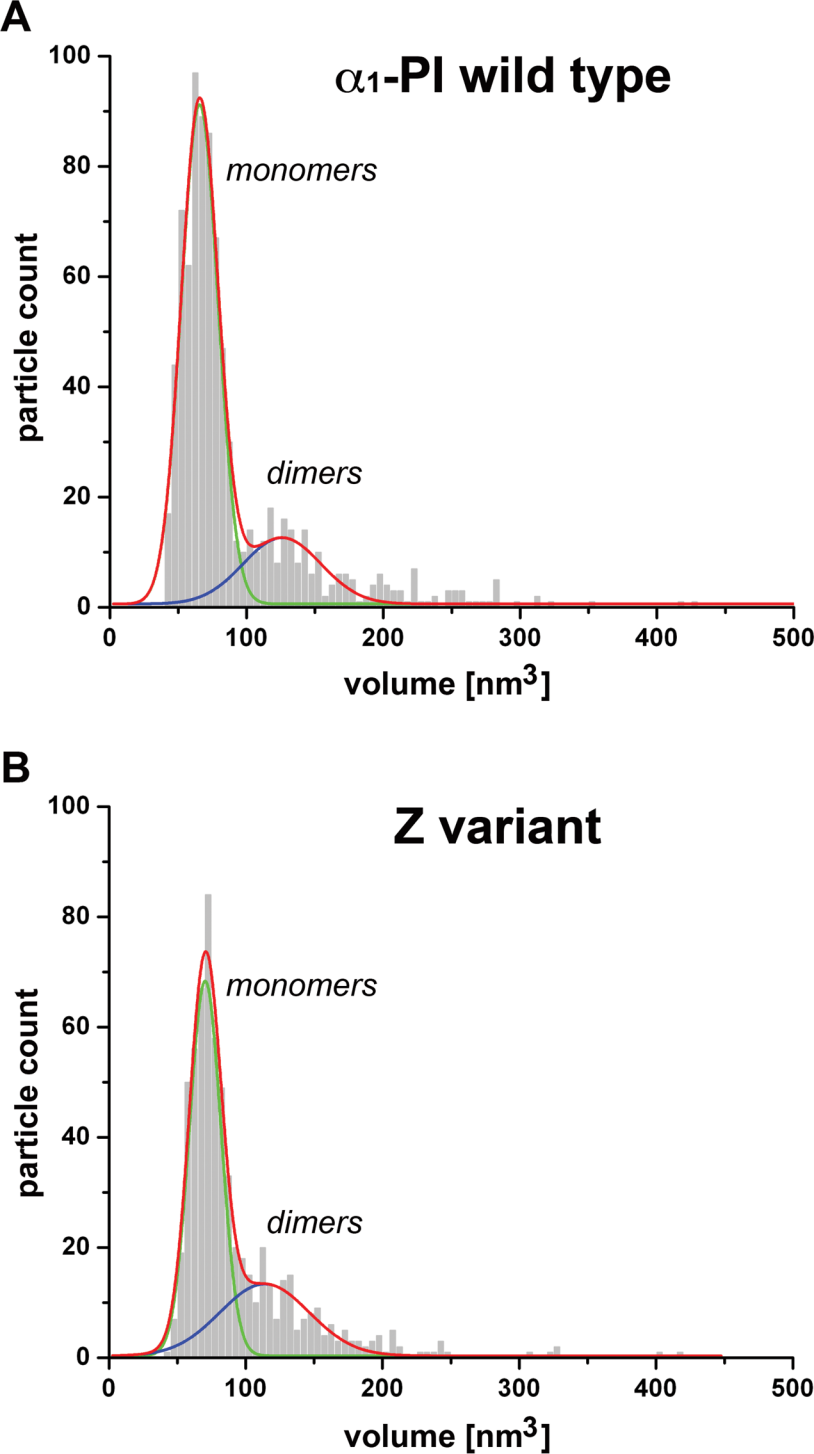
**Volume distributions of WT α**_**1**_**-PI (A) and Z variant (B) particles.** The distributions revealed a prevailing content of objects, which size was consistent with monomers. Surprisingly, the presence of larger molecules was also detected. Among the larger objects, particles with volume approximately twice as big as monomers were the most abundant. The content of dimers and bigger particles was more pronounced in the Z variant (B) than in the wild type (A). The volume of particles was calculated with the grain analysis of SPIP software. Relative count of particles is shown. Total of 847 and 639 particles were analyzed for the WT and Z variant, respectively. Histogram bin size was 5 nm. Fitted normal distribution curves are shown in green for monomers and in blue for dimers, whereas total frequency distribution is represented by red traces (OriginPro).

Fig 4 shows frequency histograms of volumes for hundreds of particles constituting all the objects imaged in the monomer preparations of wild type (847 particles; Fig 3A) and Z variant antitrypsin (639 particles; Fig 3B). Distribution of volumes confirmed that the monomers were the most populous class of particles comprising 76% and 65% of their total number in the WT and Z variant preparations, respectively. The detection of larger particles with AFM was expected based on the results of fractionation with non-denaturing PAGE (Fig 3, lanes 1 and 5). About one-third of the protein in Z variant preparations (33%±2%; n = 3) migrated significantly slower than monomers what was symptomatic of the presence of larger assemblies (Fig 3, lane 5), in a good agreement with the AFM derived data (65% of monomer, 35% slower-migrating; Fig 4). The content of larger assemblies in the WT preparation was relatively high, about 24% (Fig 4) rather than 11%±2% (n = 4) detected by electrophoretic separation (Fig 3, lane 1), a result of purposeful enrichment of the imaged WT samples in oligomers, as described above.

Cluster analysis of the histograms revealed that the average volume of the presumably monomeric particles in the major peak was 66 nm^3^ ± 12 nm^3^ (n = 609 particles) for the WT, whereas Z variant was slightly larger reaching 70 nm^3^ ± 10 nm^3^ (n = 424 particles). A tail representing larger particles followed the peak corresponding to the monomers. Therefore, the frequency for both the samples (red traces in Fig 4) as probed with the Shapiro-Wilk test did not meet conditions of the normal distribution. The peak analysis function of the OriginPro software found a population of molecules that were nearly twice as big as the monomers, likely corresponding to dimeric particles. Their size was approximately 124 nm^3^ and 114 nm^3^ for WT and Z mutant, respectively. These values were somehow lower than sums of respective monomer volumes, about 132 nm^3^ and 140 nm^3^. Such phenomenon was fully expectable since in a putative dimeric particle, areas of surface of monomeric units would not be accessible for interactions with the AFM probe and therefore do not contribute to tip broadening effect or water layer volume. The distribution of the identified populations of monomers and dimers individually satisfied the conditions of the normal distribution, marked as green (monomers) and blue (putative dimers) traces on the frequency histograms in Fig 3. Occasionally, particles larger than the dimers were also spotted, but they were particularly infrequent in the WT samples.

### AFM imaging reveals diverse topography of α_1_-PI polymerized particles

**Fig 5:**
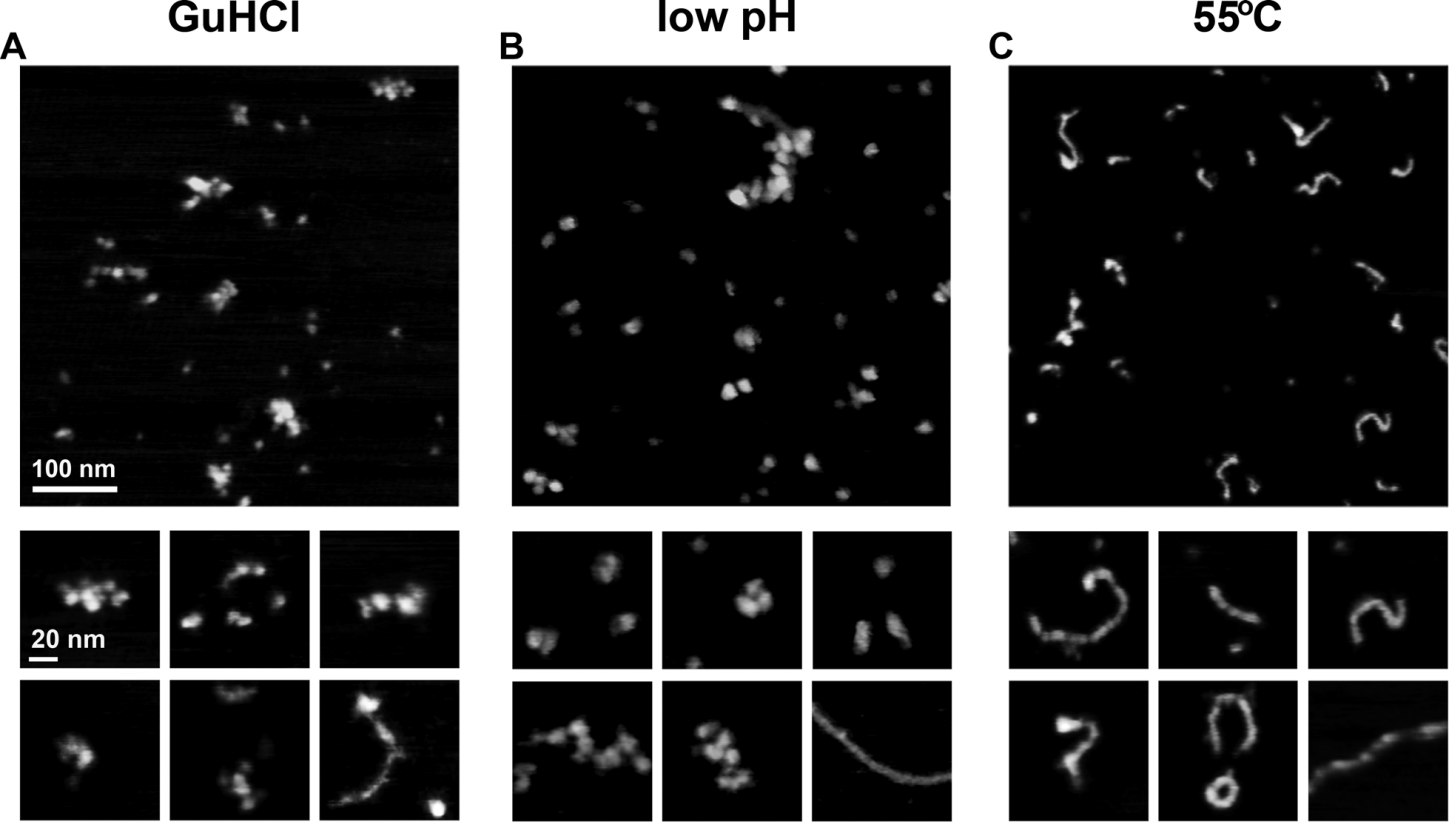
*In vitro* polymerization of led to formation of structurally diverse oligomers observed with AFM. Top panels: AFM height images of representative fields of WT α_1_-PI particles polymerized by: (A) incubation with 1.4M GuHCl (37.5 hrs, 25°C, pH = 7.4); (B) exposure to a low pH (pH = 4.1, 2 hrs, 25°C); (C) treatment with the elevated temperature (55°C, 4 hrs, pH = 7.4). Bottom panels: arrays of zoomed-in images of oligomers representative for each polymerization method.

To gain a better insight into morphological diversity of serpin polymers, we analyzed WT antitrypsin polymerized *in vitro* with three different methods: treatment with GuHCl, incubation at low pH, and exposure to 55°C. Fractionation of the *in vitro* polymerized preparations with non-denaturing PAGE revealed the presence of multiple oligomer species with distinct migration patterns (Fig 3, lanes 2–4). For each polymerization condition images of at least five distinct fields with at least fifty presumed oligomer particles were analyzed. As demonstrated in Fig 5, all the methods produced numerous large particles that were substantially bigger than monomers and dimers presented in Fig 1. The large particles were stable to the scanning conditions and did not move or disassemble during several consecutive scans. Besides the apparent oligomers, all the images still contained many small particles, indistinguishable from molecules identified earlier as monomers and dimers (Fig 1). Interestingly, the types of detected oligomers were strongly dependent on the conditions of *in vitro* polymerization. The presence of relatively short and compact particles built with tightly packed smaller units was a hallmark of samples incubated with GuHCl (Fig 5A) or at a low pH (Fig 5B). The units were consistent with the size of monomers or significantly larger, with the latter length varying from 15 nm to 20 nm. In particular, the incubation with GuHCl induced formation of clusters of large globular units (Fig 5A). The total length of clusters ranged from about 30 to 80 nm and width reached up to about 20 nm. Single cases of linear oligomers of a rather irregular width were also detected, represented by the bottom-right panel in the gallery of zoomed-in images of particles generated with GuHCl (Fig 5A). Clusters similar to those in Fig 5A were imaged in preparations treated with low pH, however they included twisted “large beads packed on a string” structures exceeding 100 nm in length (Fig 5B). Again, the thin linear oligomers were seldom present but in this case they showed a much more uniform width throughout their length (compare the bottom-right panels in the respective galleries in Fig 5A and 5B). In contrast, images of monomers subjected to the elevated temperature included numerous linear particles and very few relatively short “large beads on a string” structures (Fig 5C). No large, twisted, or irregularly shaped clusters comparable to those presented in Figs 1A and 4B were found. The linear and sometimes circular oligomers were relatively thin with a constant width reaching in most cases no more than 10 nm (Fig 5C). Based on measurements conducted on 118 linear particles the average length of the oligomers was 33 nm and the median was 49 nm, with the maximum length reaching 214 nm, and with 12% of the oligomers exceeding 90 nm in length. Most of the oligomers had a clear metameric structure suggesting their construction from similar units. The mean length of units selected from several randomly chosen thin linear oligomers was 7.1 nm ± 1.0 nm (n = 30 units) and closely resembled monomers. Some particles were capped at one end with a bulbous assembly (example: last panel in the zoom-in gallery in Fig 5A) that may represent either a structurally distinct oligomer cap or a collapsed linear part.

**Fig 6:**
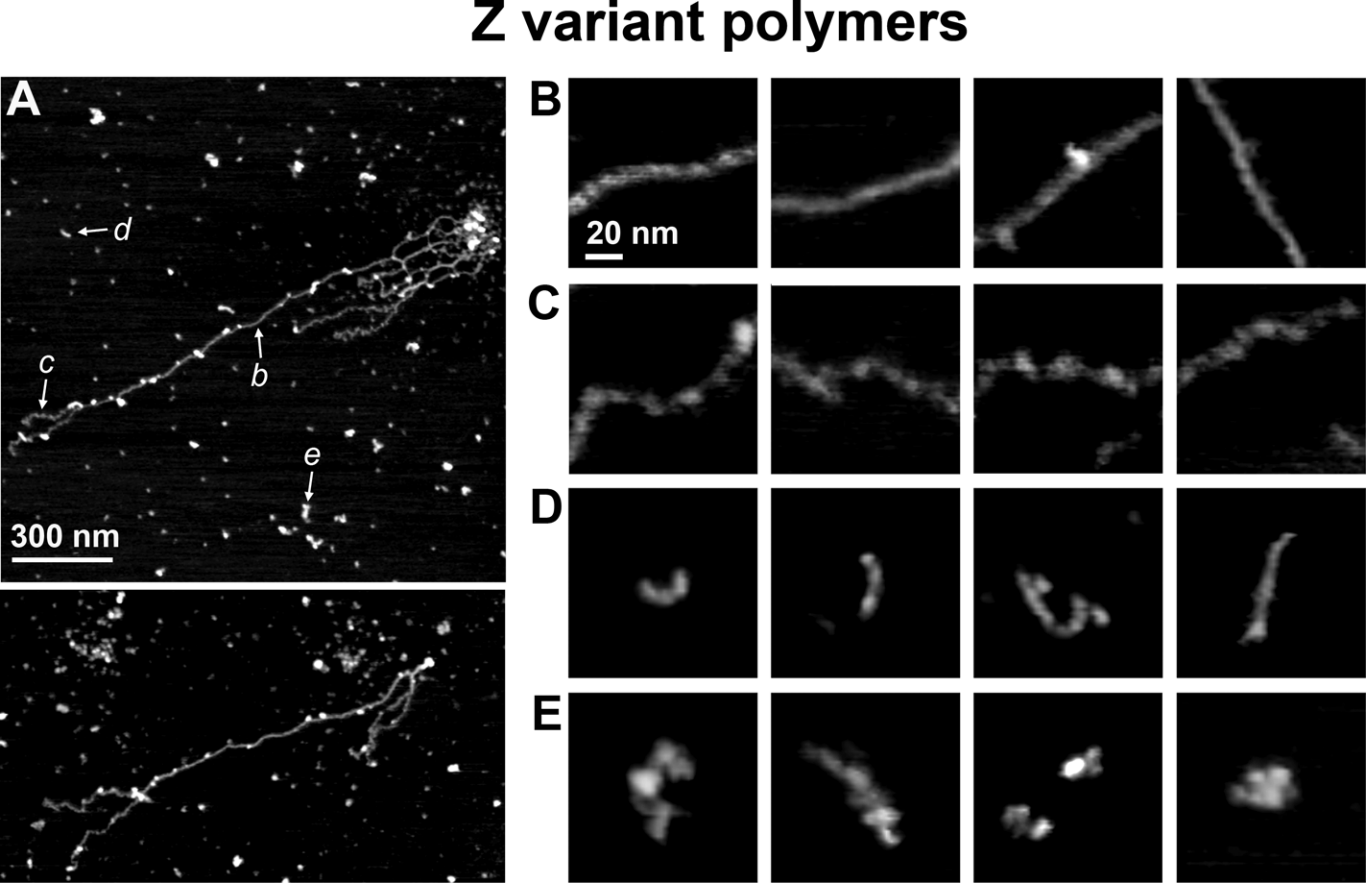
AFM height images of human Z mutant α_1_-PI preparations obtained from a PiZ mouse liver. (A) Images of two representative fragments of fields with α_1_-PI particles. The presence of long, linear, often tangled strands of polymers is striking. Arrows in the top image point at the types of particles presented in zoomed-in B to E panels on the right. The examples are marked b to e, respectively. (B)–(E) galleries of zoomed-in images of diverse polymer and oligomer types. In particular: (B) fragments of long polymer fibers with straight, smooth and compact arrangement of units; (C) fragments of long polymer fibers with grainy appearance and loose arrangement of well discernible units; (D) linear, short and thin oligomers with compact arrangement of units; (E) oligomers built from large globular units arranged as “large packed beads”.

Finally, we analyzed images of the antitrypsin Z mutant particles isolated from a liver of the transgenic PiZ mouse [25]. This particular mouse model faithfully recapitulates a slowly progressing chronic liver injury associated with accumulation of Z polymers [26]. For the imaging we used Z mutant preparations of sonicated insoluble fractions isolated from the mouse liver homogenates containing highly purified α_1_-PI protein (S1 Fig). This technique has been shown previously as very effective in isolating Z mutant polymers [22]. AFM imaging revealed that the mutant samples in terms of their topography and size contained a highly diverse population of particles (Fig 6). Similarly to the *in vitro* polymerized samples, the particles isolated from a mouse liver were stable to the scanning conditions. Monomeric and dimeric particles were still abundant. However, the most prominent feature of the Z mutant preparation was the presence of often-tangled fibers of relatively uniform width of no more than 10 nm and of continuous length exceeding 1 μm (Fig 6A). Analysis of zoomed-in sections of these polymers revealed that they were built from units consistent in size with the previously imaged monomers (compare galleries of zoom-ins in Figs 1B and 6B–6E). Interestingly, we noticed the presence of two types of unit arrangement in the long fibers, presented in Fig 6B and 6C. In the first type (Fig 6B), the fibers were straight and smooth, with tightly packed small units. The average length of units was 7.7 nm ± 1.0 nm (n = 50 units in randomly chosen polymer fragments). In the second type (Fig 6C), the fibers had a grainy and often wavy appearance, with loosely packed units, with approximate unit length of 9.2 nm ± 1.3 nm (n = 45). In addition to these very long fibers, the imaged fields contained short smooth strands (Fig 6D) and also clusters of globular particles, sometimes arranged in a “large beads packed on a string” fashion (Fig 6E). No strands forming higher order fibrils were found in the images.

We attempted to compare topographies of particles detected in the PiZ mouse liver preparation to the particles identified in the WT samples polymerized *in vitro*. We found striking similarities between images of certain types of WT and Z variant oligomers and polymers. The short smooth strands of Z polymers (Fig 6D) were very similar in their topographies to WT linear oligomers promoted by the elevated temperature (Fig 5C). The arrangement of units in these strands was undistinguishable from that of the long smooth fibers formed by the Z variant (Fig 6B). Clusters and short strings of large globular units, resembling particles detected in the WT antitrypsin sample polymerized with GuHCl (Fig 5A) or under low pH conditions (Fig 5B), were also common in the Z polymer images (Fig 6E). However, the presence of very long, micrometer-range fibers was a unique feature of the PiZ mouse liver extract.

Summarizing, AFM imaging of α_1_-PI preparations revealed the presence of particles of diverse shapes and sizes, from presumed monomers to small oligomers and to very large polymers of surprisingly diverse topography. All the types of oligomers/polymers found in PiZ liver extracts seemed to have very similar counterparts in the *in vitro* polymerized wild type preparations, with the exception of the long linear Z variant strands. The images of particles were amenable to morphometric analysis, and a sample of such analysis was presented for the volumes of WT and Z variant monomer preparations.

## Discussion

### AFM imaging provides an indispensable tool for studying morphology and spontaneous oligomerization of α_1_-PI particles

Here we show the first atomic force microscopy images of the canonical serpin antitrypsin monomers, oligomers and polymers. We visualized not only the wild type protein particles, but also the most widespread disease related Z mutant. The Z variant protein has escaped crystallization attempts. To assure the most faithful representation of the outer shape of the protein particles in our images, we used the non- invasive tapping mode for imaging. Importantly, we did not apply any chemical method of protein modification to affix antitrypsin particles. Instead, we relied on electrostatic attachment of the protein dissolved in a standard buffer to the mica substrate. Also, we did not fix the protein molecules by any kind of freezing, vacuum drying, resin embedding or metal coating. Instead, we applied gentle drying preserving the water shell of protein molecules. As a result, we obtained the AFM images of WT α_1_-PI monomer isolated from human plasma comparable in shape and size with the van der Waals space-filled crystal structure model. Antitrypsin is not a large protein judging by its primary structure (394 residues), however its relatively loose tertiary structure assures dimensions of the molecule convenient for AFM imaging. The collected images showed the oblong, kidney-shaped molecules. Extraction of detailed surface topography of the monomer particles was expectably hampered by structural dynamics, obscured by a water shell and limited by a tip dimensions. However, the characteristic oblong contour clearly indicated that the particles were resting on their sides, attached to mica in one or just a few most stable poses. Judging from the crystal structure, the α_1_-PI monomer is devoid of any apical flat surfaces that would allow for a stable top-view positioning. Therefore, the exclusive side-view pose was fully expectable.

The images were amenable for the advanced analysis of multiple morphometric parameters, now in progress. Here we limited our analysis to the particle volume. This is one of the most robust morphometric parameters utilized to detect protein dimerization and capable of particle sorting into distinct polymer classes [33,34]. Interestingly, even such limited single-parameter analysis offered an important comparison between the WT and Z variant preparations. Based on the frequency analysis, majority of AFM detected WT and mutant particles were small, with the average volume fully consistent with the presumed monomer. Although a direct evaluation of topography of wild type and Z variant α_1_-PI did not identify major differences, the average volume of the mutant was slightly larger. At this stage we cannot decide whether this result represents the true volume difference or indicates a distinct binding mode to the mica. The former could result from a more loose structure of the unstable mutant protein. The latter could lead to a mutant specific pose with its exclusive tip broadening effect producing uniquely larger monomers. Further analysis of additional morphometric parameters such as height and footprint will help to get more insight into the issue.

The images of Z variant samples contained the prevailing monomer populations but also larger particles. The trend followed closely the established native polyacrylamide electrophoresis data, detecting a significant and stable population of dimers and small oligomers in preparations of Z variant α_1_-PI purified from patient’s plasma. Importantly, electrophoresis data from this and previous studies [35] also suggested that a small population of dimers/oligomers exists in the WT preparations. A faint band corresponding to dimers and traces of slower-migrating species were clearly detectable in all tested samples. We also noticed a small population of in the images. Additionally, we succeeded in a moderate enrichment with the dimers/oligomers, from 10% or less to more than 20% of the total particle count, by washing the AFM substrate with water after electrostatic attachment of the molecules to the mica surface but before “locking” them on the surface by drying. Interestingly, such enrichment had distinct effects on the WT and mutant particles. The partition of monomeric and larger species approximated by average volume of the particles did not change in the mutant, however the washing slightly favored preservation of dimers/oligomers in the WT samples. This observation has two important implications. First, we consider it unlikely that the standard washing procedure routinely used in dry-mode AFM actually induced oligomerization. Both WT and mutant preparations were treated the same way. It would be hard to explain why the Z variant molecules, known for their propensity to form dimers/oligomers, resisted the forced oligomerization, whereas the more stable WT molecules started to oligomerize. Rather, we interpret the results by preferred washing-out of the WT monomers. Therefore, the second implication of the data is that structural differences, likely including surface charge, between the WT and mutant monomers result in their distinct behavior.

The monomer populations were prevailing in both WT and Z variant samples but clearly to a different extent. The normal distribution fitting of the major peaks in frequency analysis of particle volumes helped to estimate the relative content of each oligomer. Importantly, it revealed that larger particles were more numerous in the Z variant than in the wild type samples, even if the conditions of sample preparation enriched the latter in oligomeric species. We confirmed with AFM that the Z mutant protein appears to be thermodynamically less stable, easily forming dimers/oligomers as shown previously using a battery of biophysical tests [36].

### *In vitro* and *in vivo* polymerization of antitrypsin may proceed by multiple nonexclusive mechanisms

Imaging monomers and dimers spontaneously formed in purified monomer preparations set the stage for studying α_1_-PI polymers. In the absence of crystal structure renderings, antitrypsin polymers have been subjected to extensive biochemical and biophysical studies, and to molecular modeling in order to determine the mode of polymerization. The models were supported by the X-ray crystallographic studies of the WT monomer [31], engineered dimer [37] and trimer [38] as well as crystal and NMR structures of monomers of selected mutants other than Z variant [36,39]. Available crystal structures of other canonical serpins included antichymotrypsin monomer in a non-polymerogenic delta form [40], native human neuroserpin [41] and antithrombin dimer [37]. All these studies provided significant insight into the nature of conformational metastability and, combined with biochemical and biophysical data, and with molecular simulations, allowed for development of models of serpin polymerization. The first comprehensive, “loop-A-sheet” model calls for insertion of RCL into the A β sheet of adjacent molecule [19]. The model was over the years supplemented by numerous modifications and refinements [37,42,43]. The “loop-C-sheet” and “strand 7A” models were developed for antithrombin and plasminogen activator inhibitor-1, and they proposed side-on β sheet interactions [44,45]. A more recent “domain swap” model based on the structure of a crystallized stable antithrombin dimer, postulated exchanging of strands 4A and 5A between the adjacent molecules [37]. The modification of “domain swap” model was offered after solving the crystal structure of engineered trimer of α_1_-PI, where the C-terminal domains were swapped, rather than 4A/5A strands [38]. The “loop-sheet” and “domain swap” models anticipated formation of linear strands of compact or relaxed, respectively, monomer unit packing, however always with unidirectional, head-to-tail, orientation of monomers. To the contrary, a head-to-head model with a dimer as a basic unit in the polymer structure was proposed based on the observation that the disulfide-linked head-to-head dimer of α_1_-PI polymerizes into linear chains in a tail-to-tail manner [46]. It is worth underlining that each of the models has its own, often exclusive, set of supporting biochemical, biophysical, crystal structure, and computer simulations data.

The development of elegant models should not overshadow the fact that canonical serpin polymers are notoriously difficult subjects for direct structural analysis. Because of their size and heterogeneity, polymers have been so far beyond the reach of crystallography. Direct visualization of polymers, wild type and mutant, *in vivo* and *in vitro* formed, has been limited to electron microscopy (EM) and presented linear, sometimes circular particles with “beads on a string” organization of units [19,35,47]. A loose packing of monomers arranged in the linear or circular strings suggested the “domain swap” rather than “loop-sheet” type of polymer [48], however there is no direct evidence linking the EM images with a particular model. No other types of polymers, besides occasional irregular clumps, have been recorded with electron microscopy for α_1_-PI, even if biochemical data and molecular models implied a diversity of structural organization [49]. The lack of diversity may have originated in fixing procedures, which preselected only one type of polymers. Alternatively, it may have suggested that the presumed diversity was only a feature of *in vitro* formed oligomers, or theoretical models, and had limited *in vivo* relevance. Interestingly, EM images of another canonical serpin, human proteinase inhibitor-9 (PI-9, serpin B9) polymerized at physiological temperature, showed circular aggregates assembled into elongated strand-like structures, very different from thin “beads on a string” [50].

The problems related to harsh protein fixing for electron microscopy are avoided when atomic force microscopy is used. The AFM is very well suited to image large biomolecules and has been extensively applied to study protein polymerization mechanism [33]. The technique relies on a gentle immobilization of bioparticles and on noninvasive scanning. The opportunity of real time observations of molecular phenomena such as protein folding or aggregation is an important asset. However, canonical serpins up to now were not imaged by AFM. The closest subjects of AFM studies were aggregates of ovalbumin [51]. Ovalbumin is a member of the extended serpin superfamily. It does not function as a protease inhibitor and its polymerization is based on amyloid-type hydrophobic interactions rather than reactive center loop insertions. The AFM images of ovalbumin polymers presented periodic single strand or twisted fibrils of diverse degrees of flexibility, ultimately assembling into multistranded ribbons [51]. Periodic single-strand fibrils of diverse length were abundant in our AFM images as well, especially in heat-polymerized samples and PiZ mouse liver extracts. However, we never observed higher order assemblies formed from the fibrils, and there are no data available supporting the amyloid-type polymerization of α_1_-PI or other canonical serpins. The most striking feature of our images was diversity of topographical types of oligomers and polymers, which can be roughly classified as: (a) short, linear worm-like strands, smooth but with discernible periodicity; (b) very similar in appearance to the previous type but at least an order of magnitude longer; (c) long, grainy and highly flexible strands with prominent periodicity and a loose “beads on a string” appearance; (d) compact assemblies of large units tightly packed in a short strand-like or a nearly globular particles. The types (b) and (c) were observed exclusively in the liver extracts that were expected to contain *in vivo* polymerized α_1_-PI. The types (a) and (d) were present in all preparations, albeit their abundance varied greatly depending on the sample origin. Multiple types of polymers induced by GuHCl and the elevated temperature have been reported previously using native polyacrylamide gel electrophoresis [18], and our AFM data fully support the notion. Also, we show that the thermally induced polymers are topographically similar to the long mutant polymers, although they are much shorter. This observation is in excellent agreement with the results of probing gel-fractionated polymers with 2C1 antibodies that recognize pathological and heat stimulated polymers but do not recognize those produced with GuHCl [38,52]. However, the conclusion that the type of polymers produced with GuHCl is not relevant for *in vivo* polymerization [52] may not be correct. To the opposite, the compact assemblies built from large units were present both in the GuHCl treated preparations and in the samples isolated from a liver. However, these oligomers were very prominent in former and infrequent in the latter. It is possible that the 2C1 antibodies recognize only the most abundant species among the *in vivo* formed polymers. AFM imaging, to the contrary, detects even the very rare particle species, easily overlooked by other, ensemble-type methods.

At this stage of studies any attempt to assign an AFM detected type of particle to a specific model of oligomerization would be highly speculative. However, we may point at interesting associations. For example, the “beads on a string” strongly resemble the EM detected particles and may represent the domain-swapped polymers [48]. Yet, the flexible, curved strands often mirror the EM images of polymers formed from head-to-head dimers. Interestingly, the “beads on a string” (type c) and the smooth fibrils (type b) often seemed to be fragments of the same long strands. There may be several explanations of this observation. First, the two types may be polymerized with exactly the same mechanism, potentially domain-swapping, but attach to the mica in a distinct manner. Second, there may be different variants of polymerization in the same strand, for example adjacent domain-swapped fragments may swap distinct structural segments. The presence of loop-sheet and domain-swapped sectors in the same strand is perhaps less plausible, since the two modes are expected to generate polymers of very different degrees of compactness. Alternatively, the fragments of distinct topographies may in fact belong to separate strands, which adhered to each other and aligned when attaching to the mica. Testing a variety of conditions for polymer attachment to the mica should help to distinguish between the possibilities. In general, we hypothesize that the periodic linear strands, short or long, are topographically the closest to the domain-swapped models and, in some cases, to the head-to-head model. On the other hand, the compact assemblies built from large units (type d) are harder to classify. The tight packing of units in short strand-like particles resembles the loop-sheet oligomers [48]. On the other hand, the dimensions of units, clearly larger than monomers, suggest oligomerization of dimers or trimers. An interesting possibility would be that trimers similar to those described by Yamasaki et al. [38] and bound by C-terminal domain swapping, could form oligomers of twisted linear or nearly globular structure, as observed in AFM images. We continue detailed morphometric studies of the imaged oligomers and polymers that should aid in classification of the types of assemblies. Additional AFM experiments with specific antibody labeling of particular domains of monomers in oligomers/polymers should help to assign polymerization models to the imaged categories of molecules.

In conclusion, we present here the first AFM images of the wild type and Z mutant of the archetypal serpin, antitrypsin (α_1_-PI). The noninvasive nature of AFM imaging and the opportunity to directly visualize large number of individual particles resulted in detection of a remarkable variety of oligomeric and polymeric forms of serpin. Such diversity was proposed based on biochemical, biophysical, and molecular modeling data, however no direct imaging proof had been available. Our AFM imaging suggests that all the *in vitro* produced types of oligomers have their corresponding counterparts in the preparations generated from the liver of mouse model of the human serpinopathy. This notion stays in contrast to many previous reports favoring certain *in vitro* methods, in particular heat treatment of wild type monomers, as models for the *in vivo* polymerization. Indeed, we found that the heat polymerized wild type preparations had especially a high content of periodic linear oligomers. They were much shorter but otherwise very similar in topography to the polymer strands isolated from a liver. However, less abundant types of oligomers with diverse topographies were easily detectable in the liver extracts by employing the single molecule imaging capabilities of AFM. The described molecular diversity brings an exciting possibility that the *in vivo* polymerization of canonical serpins is achieved through various paths and by using distinct mechanisms.

## Supporting Information

S1 Fig SDS-PAGE and Western blot analysis of preparations obtained from a PiZ mouse liver. The livers were processed and the whole homogenate was separated into soluble and insoluble fractions as described in Material and Methods. For electrophoresis (7.5% polyacrylamide gel; Ready Gel BioRad), the washed pellet (insoluble fraction) was resuspended in a reconstitution buffer, dissolved and denatured in SDS-PAGE sample buffer. Separated proteins were silver stained or Western blotted and probed with specific rabbit anti-human α_1_-PI polyclonal antibodies (Biomeda). (A) SDS-PAGE (7.5% polyacrylamide gel; non-reducing), silver stained, with the position of α_1_-PI monomer (52 kDa) marked with an arrow. Lane 1: control human α_1_-PI monomer purified from plasma, 0.25 μg loaded (see also Fig 3); Lane 2: total liver homogenate, 1 μg of protein loaded; Lane 3: soluble fraction obtained from liver homogenate by centrifugation, 1 μg of protein loaded; Lane 4: insoluble fraction prepared from the same homogenate, 0.25 μg loaded. (B) Western blot probed with specific rabbit anti-human α_1_-PI afntibodies. Lanes 2, 3 and 4 correspond to total liver homogenate, soluble and insoluble fractions, respectively, as in panel A. Detection was performed with HRP conjugated goat ant-rabbit antibodies (Chemicon Int) using the ECL Plus (GE HealthCare) chemiluminescence system.
(TIF)
