# Supplementary figures and images for: AFM Imaging Reveals Topographic Diversity of Wild Type and Z Variant Polymers of Human α1-Proteinase Inhibitor
Source: PLoS One. 2016 Mar 23;11(3):e0151902. doi: 10.1371/journal.pone.0151902 (PMC4805282; doi:10.1371/journal.pone.0151902)

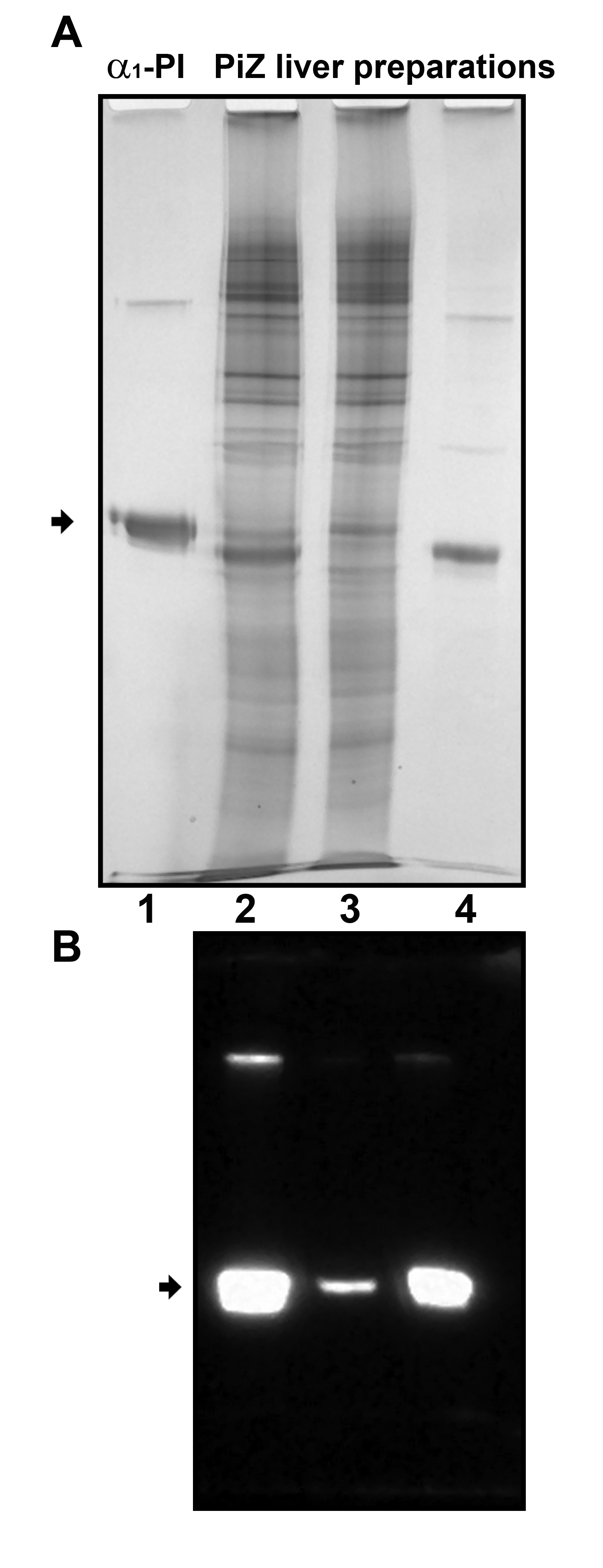

Supplement: S1 Fig — The livers were processed and the whole homogenate was separated into soluble and insoluble fractions as described in Material and Methods. For electrophoresis (7.5% polyacrylamide gel; Ready Gel BioRad), the washed pellet (insoluble fraction) was resuspended in a reconstitution buffer, dissolved and denatured in SDS-PAGE sample buffer. Separated proteins were silver stained or Western blotted and probed with specific rabbit anti-human α1-PI polyclonal antibodies (Biomeda). (A) SDS-PAGE (7.5% polyacrylamide gel; non-reducing), silver stained, with the position of α1-PI monomer (52 kDa) marked with an arrow. Lane 1: control human α1-PI monomer purified from plasma, 0.25 μg loaded (see also Fig 3); Lane 2: total liver homogenate, 1 μg of protein loaded; Lane 3: soluble fraction obtained from liver homogenate by centrifugation, 1 μg of protein loaded; Lane 4: insoluble fraction prepared from the same homogenate, 0.25 μg loaded. (B) Western blot probed with specific rabbit anti-human α1-PI afntibodies. Lanes 2, 3 and 4 correspond to total liver homogenate, soluble and insoluble fractions, respectively, as in panel A. Detection was performed with HRP conjugated goat ant-rabbit antibodies (Chemicon Int) using the ECL Plus (GE HealthCare) chemiluminescence system. (TIF) [file pone.0151902.s001.tif]
